# PET technology for drug development in psychiatry

**DOI:** 10.1002/npr2.12084

**Published:** 2020-05-28

**Authors:** Ryosuke Arakawa, Akihiro Takano, Christer Halldin

**Affiliations:** ^1^ Center for Psychiatry Research Department of Clinical Neuroscience Karolinska Institutet and Stockholm County Council Stockholm Sweden; ^2^ Takeda Development Center Japan Takeda Pharmaceutical Company Limited Osaka Japan

**Keywords:** dopamine D2 receptor, norepinephrine transporter, occupancy, positron emission tomography, serotonin transporter

## Abstract

Positron emission tomography (PET) is a non‐invasive imaging method to measure the molecule in vivo. PET imaging can evaluate the central nervous system drugs as target engagement in the human brain. For antipsychotic drugs, adequate dopamine D2 receptor occupancy (“therapeutic window”) is reported to be from 65%‐70% to 80% to achieve the antipsychotic effect without extrapyramidal symptoms. For antidepressants, the clinical threshold of serotonin transporter (5‐HTT) occupancy is reported to be 70%‐80% although the relation between the side effect and 5‐HTT occupancy has not yet been established. Evaluation of norepinephrine transporter (NET) occupancy for antidepressant is ongoing as adequate PET radioligands for NET were developed recently. Measurement of the target occupancy has been a key element to evaluate the in vivo target engagement of the drugs. In order to evaluate new drug targets for disease conditions such as negative symptoms/cognitive impairment of schizophrenia and treatment‐resistant depression, new PET radioligands need to be developed concurrently with the drug development.

## Abstract 2

PET imaging can evaluate the central nervous system drugs as target engagement in the human brain. The uptake of [^11^C]raclopride for dopamine D2 receptors decreased from (A) baseline to (B) antipsychotic administration conditions.

## Graphical abstract

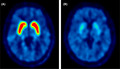

## 1 INTRODUCTION

Positron emission tomography (PET) is a non‐invasive imaging method to measure the molecule in vivo. PET imaging can evaluate biodistribution and target engagement in the human brain of central nervous system (CNS) drugs.^1^ In this short review, we firstly explain the principle of PET imaging, quantitative analysis, and calculation of the target occupancy. Then, PET occupancy studies of several antipsychotics and antidepressants are described to demonstrate how the clinical doses and the target occupancy are related.

## 2 PET RADIOLIGAND EVALUATION

### 2.1 Principle of PET

PET radioligands are, in most cases, administered to the human intravenously. The injected PET radioligand is distributed according to its characteristics such as blood‐brain barrier (BBB) permeability, affinity for target molecule, metabolism, and exertion from body The positron is emitted by positive beta decay and converts to two gamma radiations by annihilation to electron. These gamma radiations are measured outside the body using PET machine. Consequently, the degree of targeted molecule of each PET radioligand can be evaluated quantitatively. For example, [^18^F]fluorodeoxyglucose ([^18^F]FDG), which can measure the glucose metabolism, accumulates in tissues of high glucose consumption such as tumor and inflammation, and produces the large amount of gamma radiations there. By detecting the radiation, the location of the tumor and inflammation is identified.

### 2.2 Kinetic modeling

[^18^F]FDG has been the most used PET radioligand, especially in oncology field. For [^18^F]FDG‐PET, visual inspection and semi‐quantitative measurement using standardized uptake value (SUV) are useful for clinical evaluation. However, in most cases of target engagement in the brain, more accurate quantitative analysis is necessary for the evaluation of PET radioligands. When a new promising PET radioligand is developed, quantitative analysis is made to establish the most appropriate method for the PET radioligand.^2^ Arterial blood sampling and metabolite analysis in the plasma samples are performed in parallel as well. Compartment model analyses such as 1‐tissue compartment model (1TCM) and 2‐tissue compartment model (2TCM) are often applied to investigate whether the model can describe the PET dynamic data.

**Figure 1:**
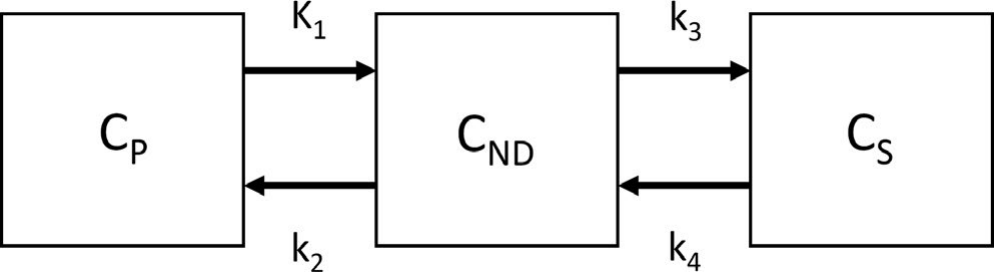
The schema of two tissue compartment model (2TCM). *C_P_*: compartment of plasma, *C*_ND_: compartment of non‐displaceable binding, *C_S_*: compartment of specific binding, *K*_1_‐*k*_4_: rate constants between compartments

In this section, as an example, we explain 2TCM as follows (Figure 1). In brief, 4 rate constants, *K*_1_‐*k*_4_, are calculated mathematically from the time‐activity curves of radioactivity in both blood and brain. Usually, binding potential (BP_ND_), which is calculated by k_3_/k_4_, is estimated as main outcome measures. BP_ND_ is defined as B_avail_/*K_d_*, as *B*_avail_ is the density of target available to bind PET radioligand in vivo and *K_d_* is the dissociation constant of PET radioligand.^3^ Consequently, BP_ND_ is a proportional value of the density of target molecule. The total distribution volume (*V_T_*), which is calculated by *K*_1_/*k*_2_ × (*k*_3_/*k*_4_ + 1), is also used. This value represents the ratio of radioactivity between brain and plasma.

Non‐compartment models are also used as alternative approach, for example, Logan graphical analysis (GA)^4^ and Ichise multilinear analysis (MA1)^5^, which allow estimating *V_T_*. GA shows a robust estimation of *V_T_* because of linear regression method, but the values decrease with increasing noise. MA1 is a modified method of GA, which aims to improve the bias.

### 2.3 Reference tissue model

**Figure 2:**
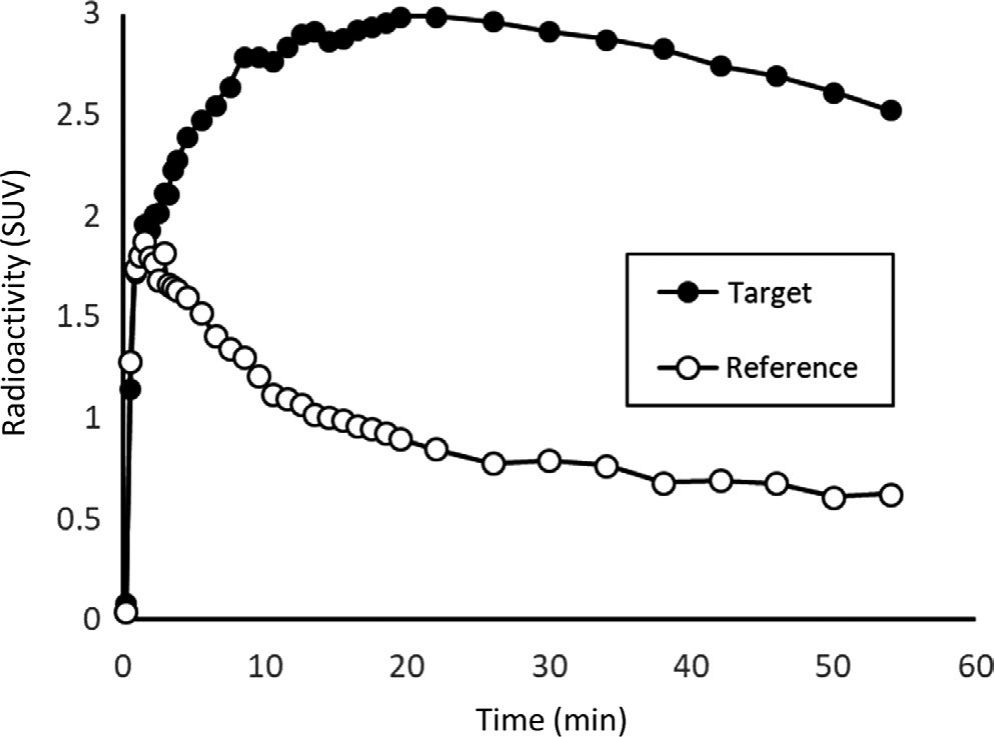
An example of time‐activity curves of [^11^C]raclopride in target region (striatum) and reference region (cerebellum). High uptake was observed in the target region compared to the reference region. SUV: standardized uptake value

Estimation of the kinetic parameter requires the arterial blood sampling to obtain the radioactivity in the blood. However, it is invasive and high burden for the participants. If there is the region without target molecule, BP_ND_ can be calculated, without arterial blood sampling, that is, using only the radioactivity in the brain. Simply, the relative difference of target and reference regions represents the BP_ND_ (Figure 2). For example, we can use the cerebellum as reference region for the evaluation of dopamine and serotonin system, and the caudate for norepinephrine system. The PET radioligands which are mentioned in this review are fit to the reference tissue models, meaning no requirement of the arterial blood sampling. Several methods have been developed as reference tissue models, for example, simplified reference tissue model (SRTM)^6^, multilinear reference tissue model (MRTM)^7^, and reference Logan^8^. SRTM is based on the two assumptions: (1) *V*_ND_ (distribution volume of non‐displaceable compartment) is the same for the target and reference tissues and (2) the kinetics in the target tissue can be fitted by a 1TCM.

## 3 TARGET ENGAGEMENT (OCCUPANCY)

**Figure 3:**
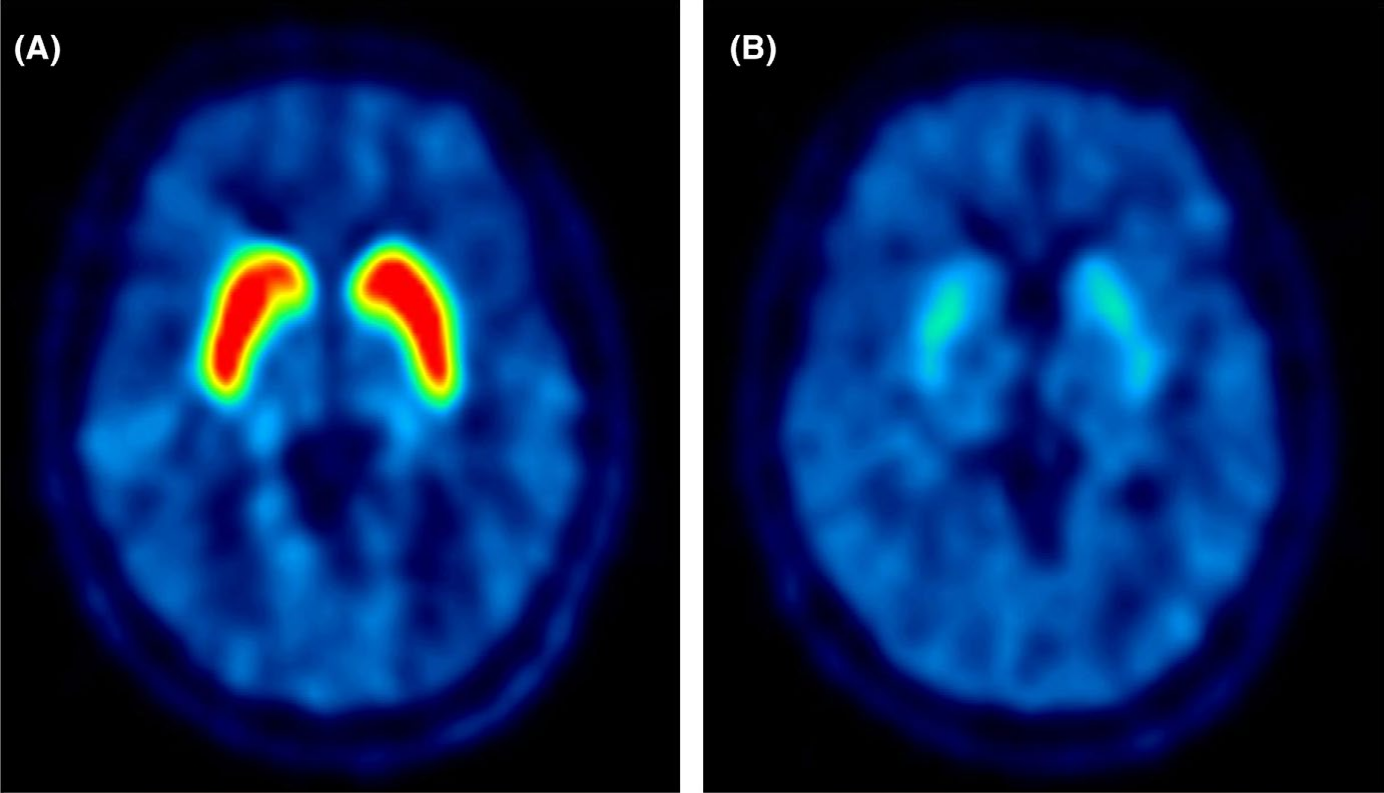
An example of PET images of (A) baseline and (B) drug administration conditions ([^11^C]raclopride). Uptake of striatum decreased after administration of the drug

In the drug evaluation, it is important to estimate its binding property of test drugs to the target molecule such as receptors or transporters. As mentioned above, BP_ND_ is the quantitative surrogate value of target molecules in PET methodology. After administration of a test drug, the BP_ND_ value decreases according to the competition model because the test drug occupies the target molecule and PET radioligand can bind to the remaining (Figure 3). Occupancy is defined as following equation: Occupancy(%) = (BP_baseline_–BP_drug_)/BP_baseline_ × 100, as BP_baseline_ is the BP_ND_ of baseline condition and BP_drug_ is the BP_ND_ of drug administration condition. It indicates %decrease of *B*_avail_ of two conditions between baseline and drug administration.

## 4 ANTIPSYCHOTICS

### 4.1 PET radioligands for dopamine D2 receptor

Main mechanism of action of antipsychotics has been the inhibition of dopamine D2 receptors. [^11^C]raclopride is the PET radioligand, which selectively binds to dopamine D2 receptors.^9^ This radioligand has ideal properties as PET radioligand such as brain uptake, affinity, time course, and mostly used for the brain PET studies. [^11^C]raclopride can measure dopamine D2 receptors in the striatum in the brain due to moderate affinity to the receptor. For the evaluation of extrastriatum, where the density of dopamine D2 receptor is quite low, [^11^C]FLB457^10^ and [^18^F]fallypride^11^, which have high affinity to the dopamine D2 receptor, are used. It would not be optimal to evaluate the striatum with these radioligands because the uptake in the striatum does not reach equilibrium during PET measurements. [^11^C]PHNO has the affinity for dopamine D3 receptor as well as dopamine D2 receptor.^12^ The binding of [^11^C]PHNO reflects the dopamine D3 receptors in the regions which has relatively higher density of dopamine D3 receptors than dopamine D2 receptors such as globus pallidum.

### 4.2 Dopamine D2 receptor occupancy and its therapeutic window

Farde et al reported the striatal dopamine D2 receptor occupancy of several antipsychotic drugs in patients with schizophrenia who responded to the drug treatment.^13^, ^14^ These studies reported that all the antipsychotics except clozapine showed over 70% occupancy. Additionally, over 80% occupancy induced the high incidence of extrapyramidal symptoms (EPS). These studies indicated that the main mechanism of action of antipsychotic was dopamine D2 receptor blockade, and EPS was related to too‐high blockade.

**Figure 4:**
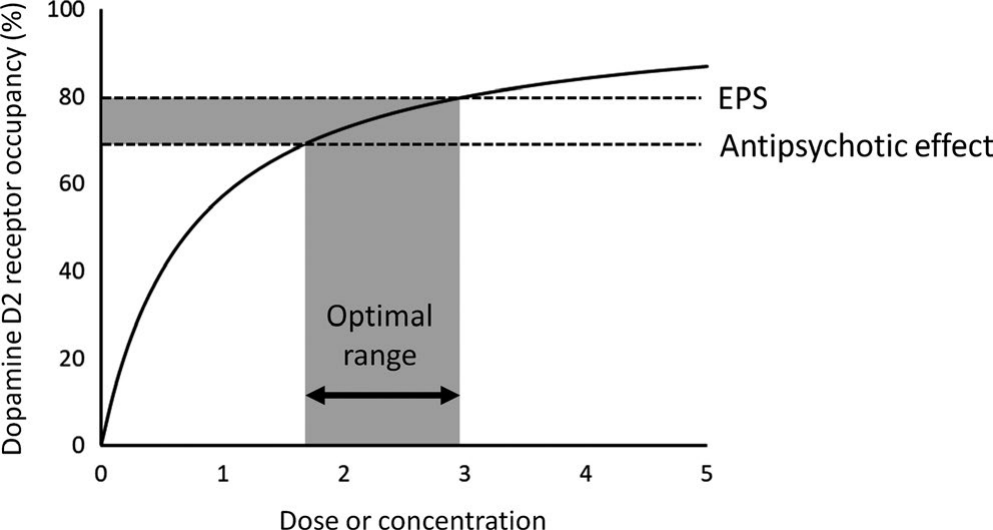
The relationship between dose or concentration and dopamine D2 receptor occupancy. Optimal range can be determined by the 70%‐80% occupancy. EPS: extrapyramidal symptoms

To test the relation of antipsychotic effect and occupancy more accurately, prospective study is needed. Nordström et al^15^ reported relationship between the dopamine D2 receptor occupancy and the clinical effect of antipsychotic in a double‐blind study. The significant relationship between the occupancy and clinical effect was observed as 70% occupancy induced 50% reduction of Brief Psychiatric Rating Scale (BPRS). Additionally, over 80% occupancy induced EPS. Kapur et al confirmed the antipsychotic effect and occupancy in a double‐blind study.^16^ They concluded that 65% was the threshold of the antipsychotic effect, and over 78% occupancy was the risk of EPS. Ziprusky et al^17^ also reported the relation between dopamine D2 receptor occupancy and antipsychotic effect. They concluded that 70% occupancy is the threshold of antipsychotic effect. Taken together, the adequate occupancy (“therapeutic window”) is thought to be from 65%‐70% to 80% (Figure 4). This concept has been widely accepted and applied to confirmation of the dose setting of so‐called second‐generation antipsychotics, such as risperidone^18^, ^19^, ^20^ and olanzapine^21^, ^22^. When first‐generation antipsychotics were re‐evaluated, it was reported that optimal dose setting of some antipsychotics might not have been made in the target occupancy's point of view.^23^

### 4.3 PET radioligands for 5‐HT2A receptor

Many second‐generation antipsychotics have the affinity for 5‐HT2A receptors in addition to the dopamine D2 receptors. Initially, [^11^C]NMSP had been used for the quantification of 5‐HT2A receptors.^24^ However, this radioligand also has the affinity for dopamine D2 receptors. Later, [^11^C]MDL 100907^25^, [^18^F]setoperon^26^, and [^18^F]altanserin^27^ have been developed as a highly selective radioligand for 5‐HT2A receptors.

### 4.4 Relationship between the clinical efficacy and 5‐HT2A receptor

Compared to the dopamine D2 receptor occupancy, there has not been clear evidence of the relationship between 5‐HT2A receptor occupancy and clinical efficacy. For example, 5mg/day of olanzapine, which is considered as lower dose than having clinical effectiveness, already showed over 90% 5‐HT2A receptor occupancy.^22^ One possible mechanism for the low risk of EPS about second‐generation antipsychotics is thought as the blockade of 5‐HT2A receptors. However, the likelihood of EPS depends on the dopamine D2 receptor occupancy, regardless of 5‐HT2A receptor occupancy.

### 4.5 Several examples of PET occupancy measurements for antipsychotics recently approved

#### 4.5.1 Paliperidone ER

Paliperidone is an active metabolite of risperidone (Table 1). Paliperidone ER is the extended release formulation of paliperidone, which is available in Japan from 2011. The dopamine D2 receptor occupancy of paliperidone ER was estimated in a part of phase II clinical trial of this drug.^28^ This is the first study in Japan using PET and occupancy data in the phase II clinical trial. Thirteen patients with schizophrenia, who were stable in the antipsychotic treatment, were involved in this study. Three doses of 3, 9, and 15 mg/day were tested. Based on 70%‐80% of striatal and extrastriatal dopamine D2 receptor occupancy, the optimal dosage was estimated to be 6‐9 mg/d. After the clinical trials, the approved dosage of paliperidone ER in Japan is 6‐12 mg/d.

**Table 1:** List of PET occupancy measurements for antipsychotics recently approved

Drug	Study	Target	PET ligand	Subject	Number	Dosage (mg)	Occupancy (%)
Paliperidone ER	Arakawa (2008)^28^	D2	[^11^C]raclopride	p	13	3‐15	54.2‐85.5
			[^11^C]FLB 457				34.5‐87.3
Blonanserin	Tateno (2013)^29^	D2	[^11^C]raclopride	p	15	8‐24	56.9‐83.7
			[^11^C]FLB 457				22.6‐83.3
	Tateno (2018)^30^	D2	[^11^C]‐(+)‐PHNO	c	6	12	60.4‐84.3
		D3					56.0‐88.7
Aripiprazole	Yokoi (2002)^31^	D2	[^11^C]raclopride	c	15	0.5‐30	22.8‐95.2
	Mamo (2007)^32^	D2	[^11^C]raclopride	p	12	10‐30	81‐94
	Gründer (2008)^33^	D2	[^18^F]fallypride	p	16	5‐30	50‐94

Note

c: control, p:patient, D2: dopamine D2 receptor, D3: dopamine D3 receptor.

#### 4.5.2 Blonanserin

Blonanserin is an antipsychotic drug highly selective to dopamine D2/D3 and 5‐HT2A receptors, which is available in Japan form 2008. Tateno et al reported the dopamine D2 receptor occupancy of blonanserin with fifteen patients with schizophrenia as part of an open‐label post‐marketing surveillance study (phase IV) in Japan.^29^ Three doses of 8, 16, and 24 mg/day were tested, and the optimal dose range was estimated to be 13‐22 mg/day. It was in line with the approved dose range of 8‐24 mg/day in Japan. Recently, dopamine D3 receptor occupancy of blonanserin was reported at similar degree as dopamine D2 receptor occupancy.^30^ However, contribution of D3 antagonism to the clinical efficacy has not fully established yet.

#### 4.5.3 Aripiprazole

Aripiprazole, which is available in Japan form 2006, has lower affinity for 5‐HT2 receptors than dopamine D2 receptors, different from many other second‐generation antipsychotics. Dopamine D2 receptor occupancy of aripiprazole showed over 80% at a low dose of 10 mg/d although the risk of EPS is quite low.^31^, ^32^, ^33^ As aripiprazole is a partial agonist for the dopamine D2 receptors, it is assumed that its intrinsic activity can avoid the excessive antagonism even at nearly full saturated target occupancy.

## 5 ANTIDEPRESSSANTS

### 5.1 PET radioligands for 5‐HTT

[^11^C]DASB^34^ and [^11^C]MADAM^35^ are well‐established PET radioligands for serotonin transporters (5‐HTT), which have high specific bindings and selectivity to estimate the 5‐HTT accurately.

### 5.2 Threshold of 5‐HTT occupancy for clinical efficacy as antidepressants

One of the main mechanisms of antidepressant is the inhibitory effect to 5‐HTT. Meyer et al reported that clinical dose of four selective serotonin reuptake inhibitors (SSRI) (paroxetine, sertraline, citalopram, fluoxetine) and venlafaxine showed over 80% 5‐HTT occupancy in patients with depression.^36^, ^37^ Suhara et al reported that clinical dose of clomipramine and fluvoxamine showed over 80% of 5‐HTT.^38^ Lundberg et al^39^ reported that tricyclic antidepressants (TCA) and SSRIs showed over 70% 5‐HTT in patients with depression. These studies indicated that the clinical threshold of 5‐HTT occupancy for antidepressants is 70%‐80%. However, the relation between the side effect and occupancy has not been well identified. Several studies in Japanese population have confirmed similar levels of 5‐HTT occupancy by clinical doses of antidepressants (fluvoxamine, paroxetine, sertraline, escitalopram, and milnacipran).^40^, ^41^, ^42^

### 5.3 PET radioligands for NET

Norepinephrine transporter (NET) inhibition is another mechanism of action of antidepressants. Compared to 5‐HTT, useful PET ligands for NET were not available when serotonin and norepinephrine reuptake inhibitors (SNRIs) were developed. [^18^F]FMeNER‐D2, which showed suitable characteristic for the quantitative analysis of NET,^43^, ^44^, ^45^ was developed as modified PET radioligand of [^11^C]MeNER, which was previously developed with less optimal brain kinetics.^46^

### 5.4 Relationship between clinical efficacy and NET occupancy

Because the development of the appropriate PET radioligand for NET was relatively new, the investigation of NET occupancy by antidepressants has not been widely performed yet. Therefore, the relationship between clinical efficacy and NET occupancy has not been reported so far.

### 5.5 Several examples of PET occupancy measurements for antidepressants and related drugs

#### 5.5.1 Duloxetine

Duloxetine is a SNRI (in vitro affinity: 0.8 nmol/L for 5‐HTT, 7.5 nmol/L for NET), approved in Japan from 2010 (Table 2). 5‐HTT occupancy by 5‐60 mg of duloxetine was examined using [^11^C]DASB in phase I clinical study in Japan.^47^ Based on the occupancy results, 40 mg/d or higher was recommended as the doses for further development. The approved dose range is 40‐60 mg/d in Japan. This is the first study in Japan, in which 5‐HTT occupancy was measured in the phase I clinical trial.

**Table 2:** List of PET occupancy measurements for antidepressants and related drugs

Drug	Study	Target	PET ligand	Subject	Number	Dosage (mg)	Occupancy (%)
Duloxetine	Takano (2006)^47^	5‐HTT	[^11^C]DASB	c	12	5‐60	35.3‐86.5
	Moriguchi (2017)^48^	NET	(S,S)‐[^18^F]FMeNER‐D2	c	8	20‐60	13.6‐46.7
Venlafaxine ER	Meyer (2004)^36^	5‐HTT	[^11^C]DASB	p	4	75	83.7 (mean)
	Voineskos (2007)^49^	5‐HTT	[^11^C]DASB	p	4	225‐450	85.8 (mean)
	Lundberg (2012)^39^	5‐HTT	[^11^C]MADAM	p	3	150‐300	71‐80
	Arakawa (2019)^50^	NET	(S,S)‐[^18^F]FMeNER‐D2	p	12	37.5‐300	8‐61
Milnacipran	Nogami (2013)^42^	5‐HTT	[^11^C]DASB	p	6	50‐200	33.0‐61.5
		NET	(S,S)‐[^18^F]FMeNER‐D2	p	6	25‐200	25.3‐49.9
Nortlyptyline	Sekine (2010)^51^	NET	(S,S)‐[^18^F]FMeNER‐D2	c	6	10‐75	10.0‐48.3
	Takano (2014)^52^	NET	(S,S)‐[^18^F]FMeNER‐D2	p	10	75‐200	50‐70
Tramadol	Ogawa (2014)^53^	5‐HTT	[^11^C]DASB	c	5	50‐100	29.9‐59.8
Quetiapine XR	Nyberg (2013)^54^	NET	(S,S)‐[^18^F]FMeNER‐D2	c	9	150‐300	2‐54
	Yatham (2018)^55^	NET	(S,S)‐[^11^C]MRB	p	10	195 (mean)	22.4 (mean)

Note

c: control, p: patient, 5‐HTT: serotonin transporter, NET: norepinephrine transporter.

Recently, Moriguchi et al reported that 20‐60 mg of duloxetine induced 30%‐40% NET occupancy in healthy subjects.^48^ These values are lower than 5‐HTT occupancy of same doses. Optimal relationship of combination of 5‐HTT and NET occupancy has not been fully established.

#### 5.5.2 Venlafaxine ER

Venlafaxine is also a SNRI (Ki: 82 nmol/L for 5‐HTT, 2480 nmol/L for NET), available in Japan from 2015. Meyer et al reported that 75 mg of venlafaxine ER showed over 80% 5‐HTT occupancy.^36^ Voineskos et al reported that over 225 mg of venlafaxine showed around 90% 5‐HTT occupancy.^49^ Lundberg et al^39^ reported that 150‐300 mg/d of venlafaxine showed 70%‐80% 5‐HTT occupancy. Although there is some variability of the 5‐HTT occupancy among the reports, the recommended doses of venlafaxine in Japan, 75‐225 mg, correspond to similar level of 5‐HTT occupancy to other SSRIs.

Recently, the NET occupancy of venlafaxine ER in patients with depression was reported.^50^ Relatively high dose (beyond 150 mg/d) of venlafaxine ER significantly blocked the NET (32%–61%). The relation between 5‐HTT and NET occupancy corresponds to in vitro affinity for 5‐HTT and NET.

#### 5.5.3 Milnacipran

Milnacipran is an SNRI (Ki: 8.44 nmol/L for 5‐HTT, 22 nmol/L for NET), approved firstly in Japan. Nogami et al reported that 25‐200 mg/d milnacipran showed 25%‐50% NET occupancy in patients with depression.^42^ They also reported that 5‐HTT occupancy was 60% by 200 mg/d of milnacipran. 5‐HTT and NET occupancies are relatively comparable.

#### 5.5.4 Nortriptyline

Nortriptyline is a classical antidepressant. It has relatively high selectivity to NET over 5‐HTT (Ki: 18 nmol/L for 5‐HTT, 1.49 nmol/L for NET). Sekine et al reported that 75 mg of nortriptyline blocked 40% NET in healthy subjects.^51^ Furthermore, Takano et al^52^ reported that clinical dose (75‐200 mg/d) of nortriptyline blocked NET in around 50%‐70% in patients with depression. They concluded that the required NET occupancy for clinical effect is considered as over 50% using the reported value of effective plasma concentration of nortriptyline.

#### 5.5.5 Tramadol

Tramadol is an analgesic drug, which has opiate agonism as well as inhibitory effect for 5‐HTT and NET. Ogawa et al reported that 100 mg of tramadol showed 50% 5‐HTT occupancy in human brain using healthy subjects.^53^ The approved dose of tramadol in Japan is up to 400 mg for pain, which might induce 80% 5‐HTT occupancy. This result indicated that the high end of clinical doses of tramadol could block similar level of 5‐HTT to other antidepressants.

#### 5.5.6 Quetiapine

Quetiapine is approved as an antipsychotic drug, but anti‐depressive effect has been also reported. A possible explanation for anti‐depressive effect is that norquetiapine, a metabolite of quetiapine, has high affinity for NET. Nyberg et al reported that 300 mg of quetiapine XR blocked NET at 35% occupancy in healthy subjects.^54^ Yatham et al^55^ also reported that 300 mg of quetiapine showed clinical effect for depressive symptoms and it induced 40% NET occupancy. Both studies support that NET inhibition by norquetiapine contributes to the antidepressant effect.

## 6 CONSIDERATION FACTORS FOR PET APPLICATION

As described above, carbon‐11 and fluorine‐18 radionuclides are used for the PET radioligands. Due to the short half‐life (around 20 minutes) of carbon‐11, the site production using a cyclotron and synthesis apparatus is required for the carbon‐11 PET radioligands. In case of fluorine‐18 PET radioligands (around 110 minutes half‐life), the delivery might be available. When multiple PET measurements in one day are considered to perform such as one baseline PET measurement and occupancy PET measurement with a drug, carbon‐11 PET radioligands are feasible due to the shorter half‐life.

When the PET measurements are performed, radiation exposure from the radionuclides is needed to consider. Typical radiation dose from PET measurements is at similar level to that from computed tomography (CT) scans for the clinical usage or natural background radiation for a few years.

Duration of PET measurement is relatively long, for example, 1 to 2 hours, and the subjects have to stay still during the measurement, which may make the participants feel uncomfortable especially in the patients with psychiatric/ neurodegenerative disorders. Simplified methods which can make the measurement time shorter should be considered if possible.

## 7 CONCLUSION

PET imaging has been widely used for the evaluation of CNS drugs. Especially, the estimations of dopamine D2 receptor occupancy for antipsychotics and 5‐HTT occupancy for antidepressants have played essential roles for dose finding in clinical trials. More evidence will be required to clarify the relationship between clinical effects and NET occupancy. Recently, the clinical targets for CNS drug development have been shifted to the negative symptoms/cognitive impairment of schizophrenia, treatment‐resistant depression, and so on. These clinical targets may involve glutamate, GABA, acetylcholine, and cannabinoid systems other than classical monoaminergic systems. PET imaging, together with concurrent development of adequate PET radioligands, would become more important technique for the evaluation of such new targets.

## CONFLICT OF INTEREST

AT is an employee of Takeda Pharmaceutical Company Limited. Other authors declare no conflict of interest.

## AUTHOR CONTRIBUTIONS

All authors had the substantial contributions to the conception of the work. RA wrote the initial draft of the manuscript. All authors revised it critically and approved the final version to be published.
